# Histoplasmosis and Blastomycosis in Solid Organ Transplant Recipients

**DOI:** 10.3390/jof1020094

**Published:** 2015-06-30

**Authors:** Carol A. Kauffman, Marisa H. Miceli

**Affiliations:** 1 Division of Infectious Diseases, Department of Internal Medicine, Veterans Affairs Ann Arbor Healthcare System, 2215 Fuller Road, University of Michigan Medical School, Ann Arbor, MI 48105, USA; 2 Division of Infectious Diseases, Department of Internal Medicine, University of Michigan Medical School, Ann Arbor, MI 48105, USA; E-Mail: mmiceli@umich.edu

**Keywords:** histoplasmosis, blastomycosis, endemic mycoses, solid organ transplantation, liposomal amphotericin B, azoles

## Abstract

Histoplasmosis and blastomycosis are geographically restricted dimorphic fungi that cause infection after the conidia produced in the mold phase are inhaled into the lungs. In the lungs, at 37 °C, these organisms undergo transformation into the yeast phase. In transplant recipients, infection can occur by exposure to the mold in the environment, by reactivation of infection that had occurred previously and had been controlled by the host until immunosuppressive medications were given post-transplantation, and finally by transmission from the donor organ in the case of histoplasmosis. In transplant recipients, disseminated infection is common, and pulmonary infection is more likely to be severe than in a non-immunosuppressed person. Diagnosis has been improved, allowing earlier treatment, with the use of rapid antigen tests performed on serum and urine. Initial treatment, for all but the mildest cases of acute pulmonary histoplasmosis, should be with a lipid formulation of amphotericin B. After clinical improvement has occurred, step-down therapy with itraconazole is recommended for a total of 12 months for most transplant recipients, but some patients will require long-term suppressive therapy to prevent relapse of disease.

## 1. Introduction

Histoplasmosis and blastomycosis are not common infections among recipients of solid organ transplants, but in this population, these infections can be devastating [1,2,3,4]. *Histoplasma capsulatum* and *Blastomyces dermatitidis* are environmental fungi that occur only in specific geographic areas. Physicians who practice in those areas usually are cognizant of the clinical manifestations of infection with these fungi, but those who practice outside these areas may not think of or easily make the diagnosis of histoplasmosis or blastomycosis. The diagnosis is most straightforward in a patient who lives in an area endemic for these fungi and who presents with acute pulmonary infection.

In immunosuppressed patients, the clinical picture may not be as clear, especially if they are living outside the area known to be endemic for these fungi. *H. capsulatum*, and to a lesser extent, *B. dermatitidis*, are able to remain quiescent after initial infection. The organism can reactivate years later when immunosuppression has occurred [5,6]. A transplant recipient who grew up in an area endemic for histoplasmosis, but has not been back to that area in many years could present with reactivation histoplasmosis. Not surprisingly, the diagnosis may be missed by physicians who are practicing in a community in which histoplasmosis has never occurred. A detailed pre-transplant history of prior areas of residence and travel is essential to knowing whether an endemic mycosis, such as histoplasmosis or blastomycosis is possible.

To compound the problem, *H. capsulatum* can be transmitted with the donor organ [7]. During maximal immunosuppression post-transplantation, reactivation occurs and infection becomes widely disseminated unless caught early. In a non-endemic area, there would likely be no hint that histoplasmosis is the diagnosis unless the source of the donor organ is considered.

*H. capsulatum* and *B. dermatitidis* cause disease in healthy people, but in transplant recipients are more likely to cause severe disseminated infection. The severity of infection depends on the type of exposure and the number of conidia inhaled, but also depends on the immune response that the host can mount. Solid organ transplant recipients who remain on an aggressive immunosuppressive regimen after the immediate post-transplant period remain at high risk for infection during this entire time.

We will review the epidemiology, clinical manifestations, diagnosis, and treatment of histoplasmosis and blastomycosis in solid organ transplant recipients.

## 2. Histoplasmosis

### 2.1. Mycology and Pathogenesis

*H. capsulatum* is a dimorphic fungus that is a mold in the environment and when grown in the laboratory at 25–30 °C [5]. Two different types of conidia, the infectious elements, are produced. The larger conidia (tuberculate conidia) are 8–15 µm in diameter, have a thick wall, and distinctive projections on the surface. These structures provide a strong hint to the identity of the fungus. The smaller conidia, 2–4 µm, are the infectious form because their size allows them to be inhaled into the alveoli. At body temperature and at 35–37 °C in the laboratory, the organism is a tiny, 2–4 µm, oval budding yeast.

After the small conidia are aerosolized into the lungs, they are phagocytized by alveolar macrophages. Within macrophages, the conidia transform to the yeast phase, and then, in the cells, disseminate widely through the lymphatics and the bloodstream. Although dissemination occurs in most infections, it is rarely symptomatic. When T lymphocytes become sensitized to the antigens of *H. capsulatum* and produce cytokines that arm the macrophage to kill the intracellular organism, the infection can be contained [8]. However, in immunosuppressed hosts, cell-mediated immunity may not develop, the organism remains viable within macrophages, and disseminated infection ensues.

### 2.2. Epidemiology

*H. capsulatum* is found in the soil in the Mississippi and Ohio River valleys, but is also found in microfoci along the eastern coast [9]. Additionally, it is commonly found throughout Central America, and periodically is reported from focal areas in Africa and Southeastern Asia. The organism grows best in soil that has a high nitrogen content, which is frequently found under trees used by birds for roosting or in caves that provide nesting places for bats [5]. Point-source outbreaks associated with activities, such as demolition of old structures and large landscaping projects, have led to infection in many people, but most cases in transplant patients appear to be sporadic and not directly related to specific high-risk behaviors leading to exposure.

The five-year transplant-associated surveillance network (TRANSNET) study that collected data from 15 transplant centers in the U.S. between 2001 and 2006 established a 12-month cumulative incidence rate for histoplasmosis among solid organ transplant recipients of 0.1% [3]. They reported on a total of 48 cases of histoplasmosis, noting that the occurrence was bimodal, with 40% of cases occurring within the first six months, but others occurring as long as 18–20 years after transplantation. Kidney transplant recipients accounted for half the cases of histoplasmosis, and liver transplant recipients for a quarter.

In the small number of cases in which *H. capsulatum* was transmitted with a donor organ, the illness was severe and usually occurred early after transplantation during the time of maximal immunosuppression [3,7,10,11,12,13,14]. In several patients, *H. capsulatum* was found in the donor organ at the time of or immediately after transplantation, and the recipients were treated pre-emptively and successfully with antifungal agents before they developed symptoms of histoplasmosis [3,11].

### 2.3. Clinical Manifestations

In a normal host, histoplasmosis is primarily a pulmonary infection. However, in the transplant population, depending on the extent of immunosuppression, it is more likely to be a disseminated infection [10,15,16]. In a patient who is not markedly immunosuppressed, focal pneumonia can occur, presenting with fever, myalgias, dry cough, and dyspnea. More often, diffuse pneumonia with more severe dyspnea and hypoxemia is the presenting manifestation of pulmonary histoplasmosis in transplant recipients [11,15]. Acute respiratory distress syndrome (ARDS) can occur in severe cases. Mediastinal and hilar lymphadenopathy, commonly seen in acute pulmonary histoplasmosis in healthy hosts, is not seen often in transplant recipients.

Disseminated histoplasmosis involves primarily the reticuloendothelial system; liver, spleen, bone marrow, and lymph nodes are commonly affected [17]. Additionally, skin, gastrointestinal tract, adrenals, and the central nervous system (CNS) can be involved. The manifestations vary depending on the organs most affected. Fever, chills, night sweats, malaise, and weight loss are common. When the infection is severe, the patient can present in septic shock, can have symptoms and signs of adrenal insufficiency, and can develop disseminated intravascular coagulation.

Physical findings pointing toward disseminated histoplasmosis include hepatosplenomegaly, painful oropharyngeal ulcers or mass lesions, and skin lesions that may be papular, pustular plaque-like, or ulcerated. Patients with gastrointestinal tract involvement frequently have diarrhea, weight loss, and abdominal pain; endoscopy often shows diffuse involvement throughout the gut. Those patients who have CNS involvement usually have symptoms suggesting a subacute meningitis. Multiple small enhancing lesions can be seen on magnetic resonance imaging, but may be relatively asymptomatic.

### 2.4. Diagnosis

Routine laboratory studies that should prompt one to think about disseminated histoplasmosis include abnormal liver enzymes, primarily an elevated alkaline phosphatase, and pancytopenia, reflecting infiltration of the bone marrow. High serum potassium and low serum sodium occur with adrenal insufficiency, and inflammatory markers (erythrocyte sedimentation rate, C-reactive protein, ferritin) are almost always elevated.

The definitive diagnosis of histoplasmosis is made by growing *H. capsulatum* in the laboratory [5]. Appropriate specimens for culture include blood, sterile body fluids, tissue biopsies, sputum, and bronchoalveolar lavage (BAL) fluid. *H. capsulatum* grows slowly, taking as long as 4–6 weeks to grow. Even using the isolator lysis-centrifugation system for blood cultures, which is a more sensitive method for growing organisms such as *H. capsulatum*, the delay is often weeks. Because of the delay in establishing the definitive diagnosis, other methods must be relied upon to quickly make a presumptive diagnosis, especially in an immunosuppressed patient who is quite ill.

Histopathological identification of the typical 2–4 μm budding yeasts in tissue biopsies is a rapid means to identify a patient who has histoplasmosis. Staining the tissue with methenamine silver stain or periodic acid-Schiff (PAS) stain is necessary to visualize the small yeast structures. Occasionally, in disseminated infection, the organisms can be seen within neutrophils or monocytes on a peripheral blood smear.

Tests for circulating *Histoplasma* antigen, performed on urine and serum, increasingly are used for rapid diagnosis in immunosuppressed patients. The test detects and quantitates circulating galactomannan of the cell wall of *H. capsulatum* [18]. Sensitivity has increased with several modifications of the test, and both serum and urine should be tested [19,20]. Antigen testing of BAL fluid can be helpful in patients who have possible pulmonary infection [21]. False positive test results occur with other endemic mycoses, most notably blastomycosis [18]. Interestingly, patients who have disseminated histoplasmosis and who have a high burden of organisms can have a false positive *Aspergillus* galactomannan result because of similarity of the cell wall polysaccharides of the two organisms [22]. However, patients who have invasive aspergillosis have not been reported to have a false positive *Histoplasma* antigen test.

In immunosuppressed hosts, who often cannot mount a good antibody response, antibody tests for *H. capsulatum*—complement fixation and immunodiffusion—are not very sensitive. In our opinion, these tests should be ordered, and if positive, may be helpful, but cannot be relied upon for diagnosis.

### 2.5. Treatment

Most transplant recipients should be treated initially with amphotericin B. Moderate to severe disseminated or pulmonary histoplasmosis in all patients should be treated with amphotericin B [23]. In transplant recipients, even for those who have less severe disease, consideration should be given to beginning therapy with amphotericin B. Within a short time (1–2 weeks) step-down therapy to an azole almost always can be accomplished. For the uncommon presentation of mild localized acute pulmonary histoplasmosis in a transplant recipient, an azole can be given as primary therapy.

Lipid formulations of amphotericin B have supplanted amphotericin B deoxycholate in many medical centers because the lipid formulations are less nephrotoxic. For histoplasmosis, liposomal amphotericin B (AmBisome) is preferred because of data in AIDS patients with disseminated histoplasmosis that showed, not only less toxicity, but also increased efficacy [24]. The dose is 3–5 mg/kg given intravenously (IV) IV daily. *H*. *capsulatum* remains very susceptible to amphotericin B, and improvement often occurs within the first 7–10 days. After the patient is clinically improved, therapy can be changed to an azole to continue the course of therapy.

If the patient has CNS involvement, the dose of liposomal amphotericin B should be 5 mg/kg daily. This agent should be continued for 4–6 weeks before changing to an azole.

The azole of choice for the treatment of histoplasmosis is itraconazole [23]. This agent is used for step-down therapy after initial amphotericin B treatment and for initial therapy in transplant recipients with mild localized acute pulmonary infection. The loading dose is 200 mg three times daily for three days, followed by 200 mg twice daily. The total length of therapy should be 12 months, but some patients may require suppressive therapy, as discussed below.

Fluconazole is a second-line agent for histoplasmosis; the dose should be 800 mg daily [23]. There is increasing experience with successful treatment of histoplasmosis with voriconazole and posaconazole, and these are preferred over fluconazole for step-down therapy when itraconazole cannot be used [25,26,27]. The dosage of oral voriconazole is 400 mg twice daily for the first day, then 200 mg twice daily. The posaconazole extended-release tablet should be used and not the oral suspension, in order to ensure better absorption. The dose is 300 mg twice daily for the first day, then 300 mg daily. For itraconazole, voriconazole and posaconazole, serum concentrations should always be measured to ensure appropriate levels (see Section on azole treatment of endemic mycoses).

Echinocandins are not active against *H. capsulatum* and should not be used for the treatment of histoplasmosis.

Because histoplasmosis has been reported to relapse in the face of ongoing immunosuppression in transplant recipients, it is prudent to continue therapy with an azole as long as the patient is markedly immunosuppressed. However, there are no firm criteria for deciding what is marked immunosuppression. Studies to guide the length of suppressive therapy for histoplasmosis have been reported in AIDS patients. In that population, suppression is recommended to continue until the patient has been treated for at least one year and their CD4 count is >150/μL for at least six months [23]. No similar criteria have been established for transplant recipients. Thus, physicians have to judge whether an individual patient is at risk for relapse of infection and whether they should remain on long-term azole therapy. The preferred antifungal agent is itraconazole, 200 mg once or twice daily.

### 2.6. Outcomes

Outcomes of histoplasmosis in transplant recipients have improved in recent years [3,4,10,11,15,16]. Mortality should be less than 10%. Worse outcomes are reported in patients in whom histoplasmosis was not thought of initially, and the diagnosis was delayed. Frequently, these patients have presented with symptoms to medical centers that are not in the geographic area in which *H. capsulatum* is endemic.

## 3. Blastomycosis

### 3.1. Mycology and Pathogenesis

*B. dermatitidis* is a dimorphic fungus that is a mold in the environment and when grown in the laboratory at 25–30 °C. At body temperature and at 35–37 °C in the laboratory, *B. dermatitidis* is converted to the yeast phase [28]. The primary mechanism of infection with *B. dermatitidis* is by inhalation of conidia from the environmental mold phase. Rarely, *B. dermatitidis* can be inoculated by a traumatic injury and cause infection [29,30]. The host response appears to be both T-cell immunity and neutrophils, which differs from that of histoplasmosis.

Pulmonary infection can be the presenting manifestation, but the organism frequently disseminates to other organs, especially skin, and patients may present with skin lesions after the pulmonary infection has cleared [31]. In a few patients, *B. dermatitidis* has been documented to reactivate years later, but this is much less common than with histoplasmosis [6]. *B. dermatitidis* has not been reported to have been transmitted by a donor organ.

### 3.2. Epidemiology

*B. dermatitidis* occurs in the U.S. in southeastern, south central, and north central states, and in Canada, the organism is found in Ontario, Manitoba, and Saskatchewan. Uncommonly, blastomycosis has been reported from isolated focal areas in Africa and Europe. The primary habitat of the organism is soil and decaying vegetation, and it is especially common along waterways [32]. Infection usually occurs in patients whose job or leisure activities take them outdoors, but in some patients no discrete exposure can be discerned.

Blastomycosis is uncommon in solid organ transplant recipients. The TRANSNET Surveillance Study reported only nine transplant recipients (five kidney, two liver, and one each pancreas and heart) with blastomycosis [3]. Disease occurred a median of six months post-transplantation, but the range extended to five years.

### 3.3. Clinical Manifestations

Transplant recipients infected with *B. dermatitidis* are more likely to develop disseminated infection and/or severe pulmonary infection than normal hosts [6,15,33]. However, in the TRANSNET study, only three of nine patients with blastomycosis had disseminated disease; the other six had only pulmonary infection [3]. Pulmonary infection in transplant recipients can sometimes remain a localized infiltrate, but more often is diffuse and may progress to ARDS [31,34]. Severe pulmonary infection is not unique to immunosuppressed patients; it is well described in previously healthy persons, as well, and presumably reflects exposure to a large number of conidia. The skin remains the most common site for dissemination; lesions are usually multiple and frequently are pustular or ulcerative. The classic verrucous lesions typical of blastomycosis are uncommon in transplant recipients. Osteoarticular structures and the prostate are other sites commonly involved with disseminated blastomycosis. CNS infection can present as meningitis or as a mass lesion [35]. Other viscera can be involved, but much less commonly than noted with histoplasmosis.

### 3.4. Diagnosis

Growth of *B. dermatitidis* in the laboratory is the definitive diagnostic test. However, it may take several weeks for the mold form of the organism to grow [28]. Appropriate specimens for culture include sputum, BAL fluid, urine, and skin. In transplant recipients, it is crucial to make a more rapid diagnosis, and thus, culture results many times are confirmatory of a diagnosis made by histopathological examination of involved tissues or by antigen assays.

The appearance of *B. dermatitidis* in tissues and body fluids is unique. With smears made from purulent material from skin lesions, calcofluor staining will readily show the large (5–15 μm), thick-walled yeast with a single broad-based bud that is characteristic of *B. dermatitidis*. A Papanicolaou stain on a cytology preparation from BAL fluid shows the characteristic broad-based budding and double-contoured cell wall quite nicely. Tissue biopsy specimens should be stained with PAS or methenamine silver stains to visualize the organism.

The *B. dermatitidis* antigen assay is performed on serum and urine by enzyme immunoassay techniques. It has proved to be useful rapid diagnostic test in patients with blastomycosis [31,36,37]. However, cross reactivity with *H. capsulatum* is exceedingly common because the two organisms share cell wall galactomannans. Commercially available antibody assays—complement fixation and immunodiffusion—for blastomycosis have not proved diagnostically useful to date.

### 3.5. Treatment

Blastomycosis should be treated initially with an amphotericin B formulation because relapses and failures have been documented in transplant recipients who received an azole as initial therapy [38]. Lipid formulations of amphotericin B have supplanted amphotericin B deoxycholate in many medical centers because of decreased toxicity. Either liposomal amphotericin B, 3–5 mg/kg daily, or amphotericin B lipid complex (ABLC), 5 mg/kg daily, can be used. Therapy can be changed to oral itraconazole, loading dose, 200 mg three times daily for three days, followed by 200 mg twice daily, several weeks after clinical improvement has occurred. The total length of treatment should be for 12 months, but in patients with persistent immunosuppression, suppressive therapy with itraconazole, 200 mg once or twice daily, is recommended [38]. It should be noted that there are no studies establishing whether suppressive therapy with an azole is effective in preventing relapse of blastomycosis.

If the patient has CNS involvement, liposomal amphotericin B, 5 mg/kg daily, is preferred to ensure higher levels in the CNS. Treatment should be for 4–6 weeks before changing to an azole.

Some patients cannot tolerate itraconazole or adequate serum concentrations cannot be achieved. The second-line agent used most often is voriconazole, and it has shown benefit in treating transplant recipients and also in patients who have CNS blastomycosis [35,39,40]. In a multi-center review of CNS blastomycosis, 9 of 10 patients were successfully treated with voriconazole [35]. Fluconazole is not as effective as itraconazole for blastomycosis but can be given if the other azoles cannot be used. To date, there is minimal experience treating blastomycosis with posaconazole [41]. Echinocandins are not active and should not be used for the treatment of blastomycosis.

### 3.6. Outcomes

The mortality of blastomycosis in transplant recipients has been reported to be 0%–36% in several series [3,6,15]. In the TRANSNET study, no patient died of blastomycosis, but two of the nine patients, both of whom had ARDS, were left with severe residual pulmonary dysfunction [3]. Deaths in other series were also ascribed primarily to severe pulmonary infection [6].

## 4. Azole Treatment of Endemic Mycoses in Transplant Recipients

### 4.1. CNS Infections

Step-down azole therapy for patients who have CNS histoplasmosis or blastomycosis is not well defined. Itraconazole, the primary azole used for the treatment of theses endemic mycoses, does not achieve adequate concentrations in the cerebrospinal fluid (CSF). Fluconazole achieves excellent concentrations in the CSF, but is less active than itraconazole against *H. capsulatum* and *B. dermatitidis*. Guidelines do not give a clear vote for one or the other for CNS infection [23,38], and both agents have been successful in individual patients.

Posaconazole is similar to itraconazole in that CSF levels are quite low. In addition, there is little experience using this agent for non-CNS histoplasmosis and blastomycosis. Thus, it cannot be recommended for treating CNS infection. Voriconazole is attractive for step-down therapy for patients with CNS infection because it achieves adequate CSF concentrations and also is active against both *H. capsulatum* and *B. dermatitidis.* It may well be the preferred agent for CNS histoplasmosis and blastomycosis.

### 4.2. Itraconazole

Better absorption is seen with the oral solution than the capsules. The oral solution should be given on an empty stomach; the capsules are given with food, and medications that decrease gastric acidity (antacids, histamine receptor blockers, proton pump inhibitors) must be avoided because gastric acid is needed for absorption. Therapeutic drug monitoring should be performed when giving itraconazole [42]. Serum levels should be obtained after steady state has been reached (usually about two weeks). Levels measured by high performance liquid chromatography (HPLC) methods are reported as both itraconazole concentration and hydroxyl-itraconazole (an active metabolite) concentration, and the two should be added together to obtain the concentration of active drug. Levels >1 μg/mL are sought [23]; we prefer to have levels >2 μg/mL. By bioassay, the levels that are reported are about five-fold higher. Drug interactions through the cytochrome P450 (CYP450) enzyme and P-glycoprotein pathways are numerous. Prior to use of itraconazole a check of all medications and interactions with itraconazole are required. This is especially pertinent in the transplant recipient in whom major interactions between itraconazole and calcineurin inhibitors lead to markedly increased levels of the immunosuppressive agent.

### 4.3. Voriconazole

Oral voriconazole has excellent bioavailability when given on an empty stomach. The loading dose is 400 mg twice daily, and then 200 mg twice daily is recommended. However, the serum concentrations of voriconazole are highly variable and hard to predict [42]. Therefore, therapeutic drug monitoring is essential when using this agent, and should be done at steady state after about 5–7 days. The preferred serum trough concentrations measured by HPLC are between 1 μg/mL and 5.5 μg/mL [43]. We prefer to have trough levels between 2–4 μg/mL in regards to efficacy. Beyond 5.5 μg/mL, toxicity, especially CNS toxicity, which includes hallucinations that can be very disturbing to patients, and hepatotoxicity increases [43]. Voriconazole is metabolized by three different CYP450 enzymes, and drug-drug interactions are many [44]. Before prescribing this agent, careful investigation of possible drug interactions is essential. Similar to itraconazole, calcineurin inhibitor levels are markedly increased when voriconazole is given.

### 4.4. Posaconazole

The original formulation of posaconazole as an oral suspension was fraught with absorption problems. The suspension had to be administered 2–4 times daily with a maximum dose of 800 mg daily; beyond 800 mg, absorption was only minimally increased. The suspension was absorbed best if given with fatty foods [45]. That all changed with the 100 mg extended release tablets, which are dosed as 300 mg twice daily for one day and then 300 mg daily after that. The tablets are usually given with food. Therapeutic drug monitoring should be followed when using posaconazole [46]. Serum levels measured by HPLC should be >1 μg/mL, but to date, there have been no adverse effects reported with levels of 2–3 μg/mL, which are now seen with use of the tablet formulation. Although posaconazole has fewer drug–drug interactions than itraconazole and voriconazole, it is a strong inhibitor of CYP3A4 and has significant interactions with many other medications, especially calcineurin inhibitors [45].

### 4.5. Isavuconazole

This newly approved broad spectrum triazole could possibly have some efficacy in the treatment of histoplasmosis and blastomycosis. *In vitro*, good activity was seen for 28 isolates of *H. capsulatum* and six isolates of *B. dermatitidis* [47]. Only seven patients with histoplasmosis and three with blastomycosis, none of which were transplant recipients, were entered into an open label treatment trial with isavuconazole [48]. Most patients showed a partial or complete response, but one patient with blastomycosis died from progressive infection. The number of patients treated is too small to recommend the use of isavuconazole for histoplasmosis or blastomycosis at this time.

## 5. Conclusions

The geographically restricted dimorphic fungi, *H. capsulatum and B. dermatitidis,* uncommonly cause pulmonary or disseminated infection in transplant recipients, but these infections are often severe and can be fatal. Early diagnosis is crucial to ensure a good outcome. More rapid diagnosis is now possible through the use of antigen tests that are performed on serum and urine and that detect cell wall galactomannans of these organisms. Initial treatment, for all but the mildest cases of acute pulmonary histoplasmosis, should be with a lipid formulation of amphotericin B. After clinical improvement has occurred, step-down therapy with an azole antifungal agent, usually itraconazole, is recommended for a total of 12 months for most transplant recipients. Patients who remain markedly immunosuppressed will require long-term suppressive therapy to prevent relapse of disease.
